# In vivo 9.4 Tesla MRI of a patient with drug-resistant epilepsy: Technical report

**DOI:** 10.1007/s00701-024-06385-4

**Published:** 2025-01-16

**Authors:** Rick H. G. J. van Lanen, Daniel Uher, Desmond H. Y. Tse, Esther Steijvers, Albert J. Colon, Jacobus F. A. Jansen, Gerhard S. Drenthen, Dimo Ivanov, Govert Hoogland, Kim Rijkers, Christianne M. Hoeberigs, Paul A. M. Hofman, Walter H. Backes, Olaf E. M. G. Schijns

**Affiliations:** 1 Department of Neurosurgery, Maastricht University Medical Centre, Maastricht, Netherlands; 2 Research Institute for Mental Health and Neuroscience (MHeNs), Maastricht University, Maastricht, Netherlands; 3 Department of Radiology & Nuclear Medicine, Maastricht University Medical Centre, Maastricht, Netherlands; 4 Scannexus, Ultra-High Field MRI Research Centre, Maastricht, Netherlands; 5 Academic Centre for Epileptology, Maastricht University Medical Centre, Heeze, Kempenhaeghe, Maastricht, Netherlands; 6 Service de la recherche et traitement d’epilepsie, Centre Hospitalier Universitaire Martinique, Fort-de-France, France; 7 Department of Electrical Engineering, Eindhoven University of Technology, Eindhoven, Netherlands; 8 Department of Cognitive Neuroscience, Faculty of Psychology and Neuroscience, Maastricht University, Maastricht, Netherlands; 9 Research Institute for Cardiovascular Diseases (CARIM), Maastricht University, Maastricht, Netherlands

**Keywords:** Epilepsy, Epilepsy surgery, MRI, UHF, 9.4T

## Abstract

**Purpose:**

In resective epilepsy surgery for drug-resistant focal epilepsy (DRE), good seizure outcome is strongly associated with visualization of an epileptogenic lesion on MRI. Standard clinical MRI (≤ 3 Tesla (T)) may fail to detect subtle lesions. 7T MRI enhances detection and delineation, the potential benefits of increasing field strength to 9.4T are explored.

**Methods:**

A 36 years old male patient with DRE evaluated for resective surgery, in which 3T and 7T MRI failed to detect any epileptogenic lesions, was submitted to a dedicated epilepsy scan protocol using T1 and T2* weighted imaging at 9.4T. Images were evaluated independently by two neuroradiologists and one neurosurgeon.

**Results:**

9.4T MRI offered increased spatial resolution and enhanced depiction of anatomical structures vital for epilepsy imaging, exemplified by regions mesio-temporal (hippocampus, amygdala), latero-temporal, insula, frontal and temporal operculum, and gray-white matter junction (precentral gyrus/frontal lobe) compared to 3T and 7T, albeit with challenges in mesial-temporal and antero-inferior temporal lobe imaging. No epileptogenic lesion was identified.

**Conclusion:**

9.4T demonstrates promise in the identification and delineation of anatomical structures and small epileptogenic lesions in patients with DRE eligible for resective surgery. Whether clinical 9.4T MRI in DRE has clinical advantages over 7T or leads to a more complete resection of the epileptogenic zone and improved seizure outcome after epilepsy surgery needs to be established.

**Supplementary Information:**

The online version contains supplementary material available at 10.1007/s00701-024-06385-4.

## Introduction

Epilepsy, a neurological disorder characterized by recurrent seizures, affects around 68 million people worldwide [31]. Drug-resistant epilepsy (DRE) poses a major problem and occurs in about 30–40% of patients with focal epilepsy [31]. An evidence-based treatment for DRE is brain surgery, whose effectiveness increases the more accurate the diagnosis and the localization of the seizure onset zone [31]. A major predictive factor for good outcome (i.e., seizure freedom) is the detection of a potential epileptogenic lesion/zone on MRI [23]. Approximately 30% of patients with DRE have no identifiable epileptogenic lesion on 3 Tesla (T) MRI scanned with a dedicated epilepsy MRI protocol, dubbed “MRI-negative” [16, 27]. In this regard, MRI scanners at ultra-high field (UHF) with field strengths of 7T and even 9.4T are of interest.

Current literature on the added value of UHF MRI, consisting of studies at 7T in patients with DRE, suggests improved detection and delineation of epileptogenic lesions [27]. This could lead to surgical treatment in patients who previously were not considered candidates for epilepsy surgery [1, 21]. Detection rates of potential epileptogenic lesions on MRI are strongly dependent on (1) application of a dedicated epilepsy protocol, (2) assessment by a neuroradiologist experienced in epilepsy imaging and (3) technical factors, such as magnetic field strength [27]. So far, the highest clinically used field strength in this patient group is 7T, showing up to 30% increase in lesion detection over 3T MRI [17, 27]. Nonetheless, using higher field strengths results in an accompanying increase in magnetic field inhomogeneities.

At ultra-high field, the B_1_+ (transmit magnetic field) inhomogeneities are exacerbated compared to lower field strengths due to the shorter radiofrequency (RF) wavelengths employed and the inherent interference effects. This effect can be mitigated by usage of multiple parallel transmit channels (pTx) [15]. Inhomogeneity issues increase with field strength and therefore pTx becomes a necessity at 9.4T [11, 25, 26]. Furthermore, RF power deposition into surrounding tissue increases for higher field strengths, as such the trade-off between signal to noise ratio (SNR), contrast to noise (CNR) and specific absorption rate (SAR) requires careful calibration and sequence design [11, 13]. The progress with in vivo imaging at 9.4T in Maastricht has been recently described in detail [11]. 

Despite the significant challenges with field homogeneity and SAR at 9.4T, the ongoing efforts at the UHF MRI in our center recently resulted in imaging a DRE patient with a suspected epileptogenic focus that was negative on 3T and 7T MRI. We hereby present image quality and evaluate suitability for pre-surgical diagnostic assessment.

## Methods and materials

The EpiUltraStudy was designed to assess the value of both 7T and 9.4T MRI for lesion detection and therapeutic impact in surgical candidates with DRE (Dutch Trial Register, ID: NTR7536). The protocol for this multicenter, prospective, therapeutic study has been published [28]. 

### Patient description

For this study, a 36-year-old man with DRE and no detectable epileptogenic lesion at 3T MRI (including postprocessing with voxel-based morphology) was recruited from the Academic Centre for Epileptology (ACE) Kempenhaeghe/Maastricht University Medical Centre+ (MUMC+). The evaluation of previous diagnostic examinations (EEG-fMRI, S-EEG, PET, MEG) suggested an epileptogenic zone in the left temporal lobe. The patient signed an informed consent for both the 7T and 9.4T MRI. Subsequent 7T MRI was negative in this patient.

### MRI-protocol

MRI acquisition was performed at Scannexus BV (Maastricht, The Netherlands), a specialized UHF MRI research facility. The scans were acquired using a Siemens Magnetom 9.4T with a head-gradient set and a 31-channel receive/16-channel parallel RF transmit head coil [20]. Development of the clinically applicable 9.4T MRI scan protocol for detection of structural abnormalities was realized as part of the study. B_0_ and B_1_ + mapping was described previously in detail and identical shimming strategy was applied during this scan as well [11]. Universal pTx pulses were designed based on an in-house produced universal pulse set obtained from healthy volunteers [8]. This was done to circumvent the process of manually calibrating each scan session, allowing for easily accessible future application.

T1w MPRAGE (magnetization-prepared rapid gradient-echo) and 3D GRE (spoiled-gradient echo) ASPIRE [5] whole brain structural scans at 9.4T were acquired, alongside the already acquired 3T and 7T images. The scan parameters of 3T MPRAGE, 7T MP2RAGE, and 9.4T sequences are summarized in Supplementary material 1. Only gradient echo, and no spin echo, type sequences were applied for reasons of SAR and availability.

### Evaluation protocol

All 9.4T images were independently reviewed in two runs by two neuroradiologists (PAMH, CMH) experienced in epilepsy-imaging, and a neurosurgeon specialized in epilepsy surgery (OEMGS). Images were reviewed for quality and potential epileptogenic lesions using a structured UHF MRI digital scoring form, allowing objective comparison between observers and scans. Mesiotemporal structures in both hemispheres were evaluated and qualitatively compared for symmetry, size, signal intensity and abnormalities such as hippocampal sclerosis (HS). Furthermore, the whole brain was evaluated for epileptogenic lesions like focal cortical dysplasia (FCD) [3]. 

Image quality between 3T, 7T and 9.4T was subjected to semi-quantitative analysis for comparison of regions of interest in MRI epilepsy evaluation, being: mesio-temporal (hippocampus, amygdala), latero-temporal, insula, frontal and temporal operculum, gray-white matter junction (precentral gyrus/frontal lobe). These regions were identified as exampled in Fig. 2. Additionally, apparent SNR and CNR were calculated for regions of interest (frontal, insular, latero-temporal, hippocampus) and compared between 3T, 7T and 9.4T [7], see Supplementary material 2.

## Results

Total scan time for the 9.4T MRI was 48 min. The patient experienced dizziness upon entering and exiting the MRI bore, but this sensation resolved within five minutes, the scan was otherwise without adverse events.

**Fig. 1:**
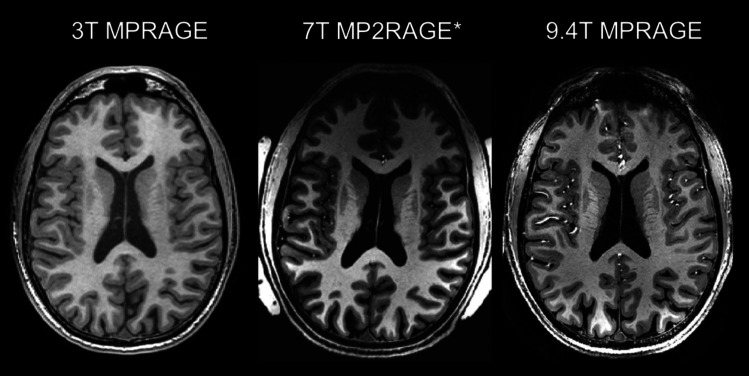
Comparison between 3T, 7T and 9.4T transverse T1w images. The 7T was obtained by multiplying the MP2RAGE unified image with the proton density weighted image. This was done to simulate the MPRAGE contrast to facilitate a more direct visual comparison

**Fig. 2:**
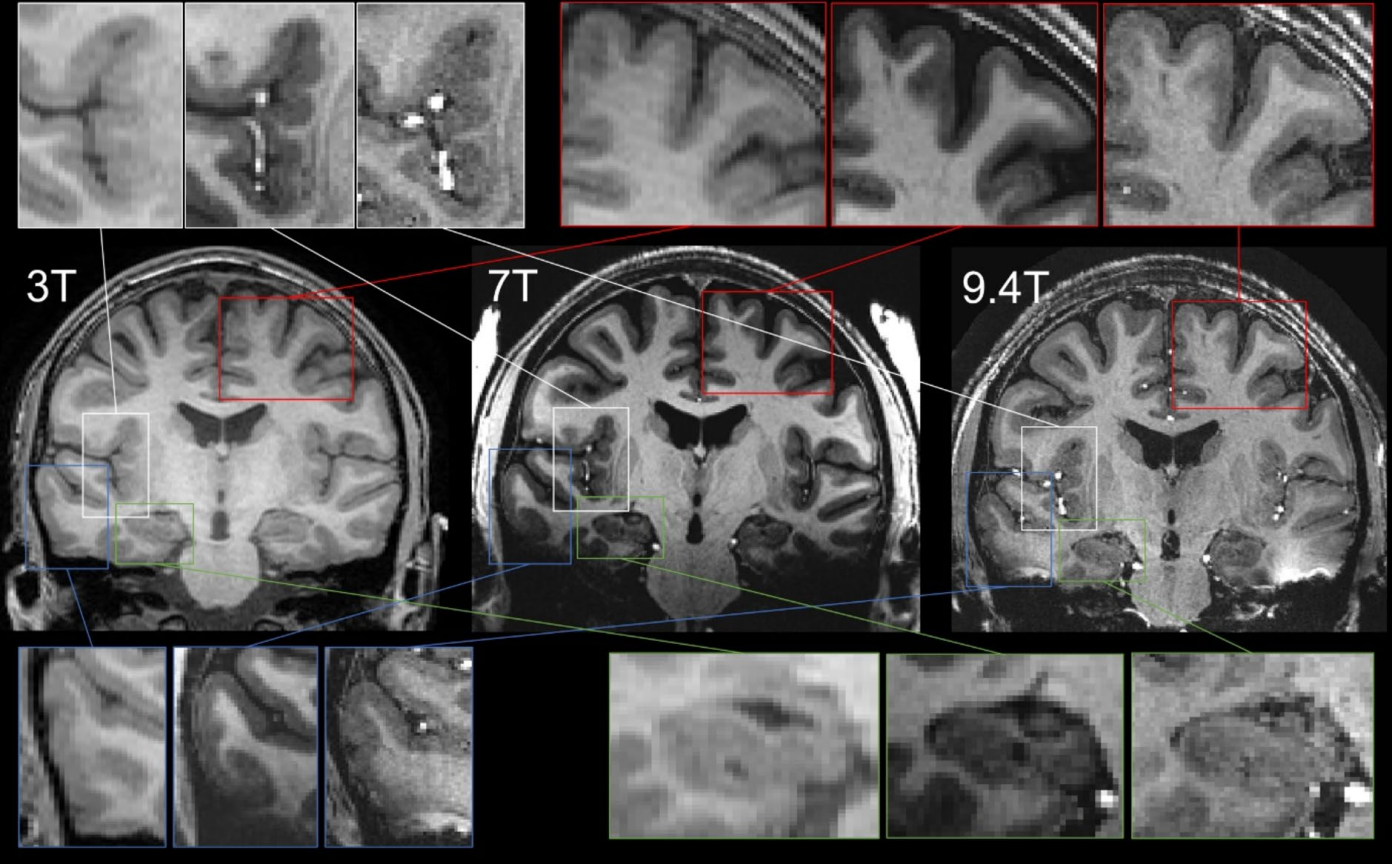
Coronal view of the 3T, 7T and 9.4T images, showing corresponding magnification of regions of interest in MRI epilepsy evaluation with top left the insular area, top right grey-white matter junction in the frontal area, bottom left lateral temporal area, bottom right hippocampus

A cubic spatial resolution of 0.6 mm was achieved in all 9.4T sequences, which was pushed from 0.9375 × 0.9375 × 0.9 mm at 3T and 0.7 mm cubic at 7T. The higher spatial resolution enabled better depiction and qualitative analysis of anatomical structures, exemplified by the regions of interest relevant for diagnostic epilepsy imaging. Figures 1 and 2 shows comparison of images at 3T, 7T and 9.4T. The grey-white matter junction, represented in the frontal lobe, insular area, and lateral temporal area, has a sharper edge with improved visualization and provides more contrast between grey and white matter with increasing field strength. The hippocampus shows more details at 9.4T. One can identify the hippocampal borders and anatomic location of the alveus and cornu ammonis band, shown in Fig. 2, features that could not be identified on 3T or 7T. There was no obvious asymmetry in hippocampal volume. Apparent SNR and CNR were calculated for regions of interest (frontal, insular, latero-temporal, hippocampus) and compared between 3T, 7T and 9.4T, see Supplementary material 2. Besides T1w MPRAGE, multi-echo gradient echo ASPIRE (T2*) sequence was acquired with different echo times (Supplementary Fig. 1). Furthermore, susceptibility weighted imaging at 9.4T was added (Supplementary Fig. 2), showing high-resolution depiction of the brain’s vasculature. For this 9.4T MRI, no structural abnormalities explanatory for the epilepsy of this patient were reported.

**Fig. 3:**
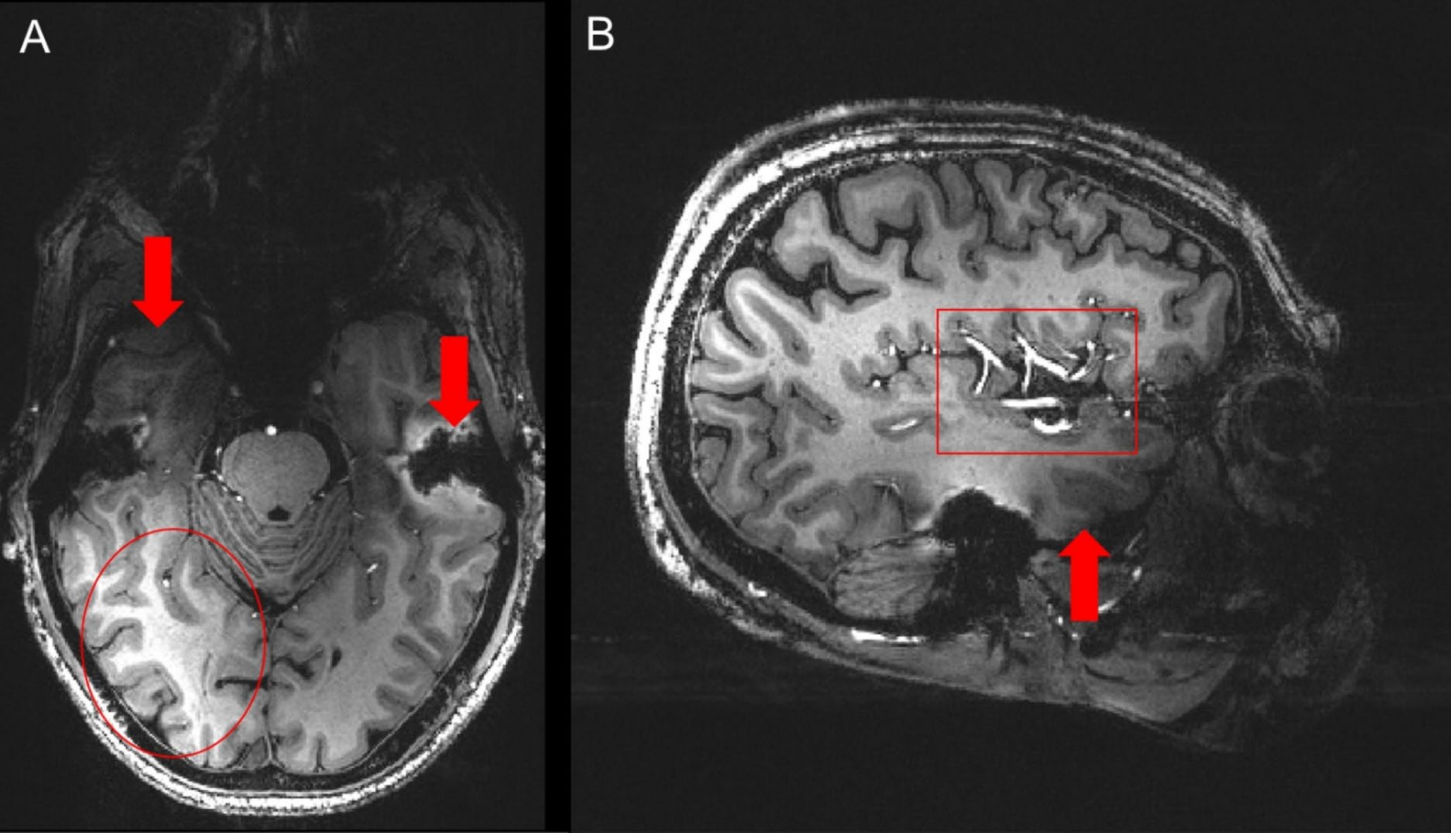
(**A**) Axial and (**B**) sagittal T1w images at 9.4T. Arrows showing signal dropout in the temporal lobe areas due to nearby air cavities, especially prominent in the anterior inferior temporal lobe. Circle showing pronounced brightness on the right temporo-occipital area, caused by proximity of coil receive elements at the posterior part of the brain, resulting in higher receive sensitivity in this region. Rectangle highlighting hyperintensity of the blood vessels

Despite field homogenization efforts, we observed signal dropouts in the temporal lobe areas, especially prominent in the anterior inferior temporal lobe (Fig. 3, arrows) and also in the inferior frontal region. This lack of signal is present in both MPRAGE and GRE images. The more pronounced brightness in the right occipital area (Fig. 3, circle) is caused by the proximity of coil receive elements at the posterior part of the brain, resulting in higher receive sensitivity in this region. Furthermore, for 9.4T MPRAGE the arterial vessels are show hyperintense compared to 3T and 7T due to the inflow of unsaturated blood (Fig. 3, rectangle).

## Discussion

UHF MRI with field strengths of 9.4T and higher are rarely performed in DRE patients and currently no cases or series have been reported in medical search engines. This is the first usage of 9.4T MRI in a patient with DRE, in which imaging at 3T and 7T was unrevealing for an epileptogenic lesion. We showed that the clinical application of 9.4T in DRE is feasible and 9.4T enabled higher spatial resolution. Visual comparison showed better depiction of the brain’s structures compared to 3T and 7T, exemplified by regions of interest in epilepsy: the cortical surface, grey-white matter junction, insular cortex, lateral temporal neocortex and white matter transient zones, and hippocampus. The rationale to increase field from 7T to 9.4T in epilepsy stems from the finding that 7T increases diagnostic gain over 3T [27]. As the next step in epilepsy imaging, we explored the role of 9.4T MRI in lesion detection as well as the possibility to increase delineation of structural lesions. Increased delineation might improve outcomes of resective surgery and is unable to be obtained by other imaging methods such as nuclear medicine. Currently, no clinically available higher field strength MRI scanners than 9.4T are available and the application in a patient with drug-resistant epilepsy represents a significant milestone in neuroimaging. Although no lesion was found in this particular case, we believe that advancements in imaging technology could benefit the entire population of epilepsy patients. This aspect is currently discussed in literature for the 7T MRI in patients who are in the trajectory for epilepsy surgery and both a diagnostic gain even as a better delineation of doubtful abnormal areas in the 3T MRI are described [10]. 

With this in mind we were able to create a 9.4T scan protocol for T1 and GRE images. The scan protocol was optimized for anatomical whole brain imaging, with efforts in optimizing B_0_ shim and B_1_ inhomogeneities to reduce signal dropouts that occasionally occur in the antero-inferior temporal lobes. While high resolution MR images with better depiction of the internal structure of the hippocampus compared to our images exist, these images have extensive acquisition times and are T2 weighted [4]. However, at 9.4T, clinical T2w imaging sequences utilize refocusing pulses which are SAR intensive. Especially turbo spin echo has many additional refocusing pulses, further enhancing SAR to levels in which clinical application of T2 weighted imaging at 9.4T is extremely challenging. This is also the reason we were not able to include T2 imaging as well, even though literature states a full ‘epilepsy MRI protocol’ should include a T2 as well [17, 22]. Using ultra-high field strengths MRI for clinical purposes requires new developments to overcome these challenges.

Field distortions interfere with encoding of contrast and spatial origin of the MR signal, commonly causing artifacts in the form of signal loss and geometric distortion, but can also contribute to ghosting, blurring and distorted excitation volumes, amongst other effects [18]. We observed a signal dropout in the temporal lobe areas. This is especially prominent in the anterior inferior temporal lobe (Fig. 3), most probably caused by the proximal bone (mastoid) air interfaces and the tympanic cavity in the temporal bone [24]. A similar effect can be also observed in the fronto-basal region due to the air-filled planum sphenoidale. These artifacts are well-documented; their mitigation remains a challenging task. For clinical evaluation of this subject, signal loss in the lower temporal lobes posed a challenge due to the suspicion for a left temporal onset.

Further increasing magnetic field strength potentially raises new safety concerns in the areas of the effects of the static magnetic field itself, increased power deposition due to the higher operating frequency, and acoustic noise. The same rigorous precautions apply at ultra-high fields as established at lower field strengths [12]. The same legal SAR-regulations are applicable to UHF-MRI as at lower field strengths and strictly adhered to [6, 11, 29]. Additionally, since the shorter RF wavelength at 9.4T commits a greater risk of RF-induced heating, patients are not allowed to have any metal implants and a metal wire behind the patient’s teeth was removed prior to the scan. Ear plugs were used alongside cushions covering the ears to reduce noise. The patients experienced dizziness upon entering and exiting the MRI’s bore, most likely caused by vestibular activation due to Lorenz forces on ion currents in the semicircular loops, which resolved in five minutes [6, 29]. Throughout the procedure, continuous communication with the subject was maintained via an intercom to monitor their well-being, especially considering the patient’s epilepsy condition. The scanning process proceeded without incident, and the subject reported no other side effects or concerns.

MRI with field strengths of 9.4T have been available for some time. Earlier publications have thus far mainly been used to image animal brains, ex-vivo imaging of sections of brain, and more recently in vivo imaging targeted at specific brain areas or even whole brain [9, 14, 19, 32]. However, clinical 9.4T imaging targeted at patients with epilepsy is currently lacking. The utilization of 9.4T MRI as an adjunctive tool in the diagnostic trajectory of patients with DRE aims to explore the potential added benefits in terms of lesion detection and delineation, possibly increasing eligible surgical candidates and improving overall seizure outcomes after epilepsy surgery [30]. 

MRI-negative patients who do undergo surgery frequently have distinct epileptogenic lesions identified post-surgery via histopathological investigations or retrospective examination of the images, especially FCD type 1 and mild malformations of cortical development [2]. Therefore, the biggest gain of 9.4T would ideally be needed in the detectability of FCD type 1 and mild malformations of cortical development, in which changes can be subtle and indistinguishable from signal averaging and partial volume effects [3]. Besides FCD, HS is a common cause of DRE and imaging studies play a crucial role in diagnosing HS. MRI can show a reduction in hippocampal volume and increased signal intensity, and is often identified in surgically removed tissue [19]. However, end-folium sclerosis, an early-stage HS, is often unrevealing on 3T or even 7T MRI [17]. At 9.4T, we showed that the hippocampal subfields are more readily identifiable compared to lower field strengths on T1 weighted images, which may aid in the detection of early-stage HS.

### Challenges and future perspectives

The main technical challenges for UHF MRI acquisition are inhomogeneities in the main magnetic field (B_0_) and radiofrequency transmit field (B_1_) and their stronger interaction with implants. Besides, suitable transmit and receive coils are not widely available as a product. Additionally, SAR limitations pose a serious challenge as well, especially rendering direct employment of all the same sequences as at 3T and 7T.

Currently 9.4T scanners are not available for most patients with epilepsy, which likely is the reason clinical 9.4T data is currently lacking. Another challenge lies in the inexperience in clinical reading/interpretation of 9.4T images, as reviewers might experience a reviewing learning curve, partly due to the lack of positive controls at 9.4T. Also, UHF might produce artefacts or geometric disturbances not seen on conventional MRI, potentially resulting in detection of non-relevant lesions (false positives).

The next step is to create a case series or cohort of patients with DRE scanned at 9.4T, who are 7T MRI negative, to establish diagnostic gain of 9.4T MRI over 3T and 7T. Additionally, to improve diagnostic yield in epilepsy lesion detection would be to expand upon the current 9.4T scan protocol and add sequences to create a full ‘epilepsy MRI protocol’ [17, 22]. Future advancements may yet overcome these issues, potentially enhancing epilepsy imaging at these field strengths and benefiting patients. Besides diagnostic gain of 9.4T, therapeutic gain also needs to be established, as well as the costs of 9.4T MRI [28]. Additionally, clinical 9.4T MRI scans might benefit from information gathered from ex vivo scanning at 9.4T in epilepsy [19]. 

As brain imaging at 9.4T might create a benefit for the field of epilepsy surgery, it also has implications for research and innovation by encouraging ongoing scientific exploration and technological advancements. Despite many years of basic and clinical research, the pathophysiology of epilepsy has not sufficiently been elucidated. Besides expansion of the cohort of eligible patients for epilepsy surgery, development of treatment alternatives is desperately needed and may be aimed at yet unraveled mechanisms in the pathophysiology of epilepsy. The knowledge gained from improved imaging of the human brain can contribute to a deeper understanding of the (patho-) physiology of the brain. These developments not only benefit epilepsy patients, but also have broader implications for neurology and neuroscience as a whole.

## Conclusion

This is the first usage of 9.4T MRI in a patient with DRE. The obtained images show an improved spatial resolution and consequently better visualization of anatomical structures compared to 3T and 7T, exemplified by regions of interest characteristic for epilepsy imaging. While 7T has been shown to increase lesion detection in epilepsy, the role of 9.4T has to be established, but demonstrates promise in the identification and demarcation of small epileptogenic lesions. The possibility of ex vivo UHF MRI on resection specimens and the correlation with histopathology may create insight into potential radiological biomarkers. Beyond epilepsy, the advancements in the field of MRI hold potential for other neurological diseases and innovative neuroscience research.

## Supplementary Information

Below is the link to the electronic supplementary material.

ESM 1 (DOCX 1.43 MB)

## Data Availability

available upon reasonable request.

## References

[1] Baumgartner C, Koren J, Britto-Arias M, Zoche L, Pirker S (2019) Presurgical epilepsy evaluation and epilepsy surgery. F1000 Res 8:1818 10.12688/f1000research.17714.1 PMC6820825 31700611

[2] Bien CG, Szinay M, Wagner J, Clusmann H, Becker AJ, Urbach H (2009) Characteristics and surgical outcomes of patients with refractory magnetic resonance imaging-negative epilepsies. Arch Neurol 66(12):1491–1499 20008653 10.1001/archneurol.2009.283

[3] Crino P (2015) Focal cortical dysplasia. Semin Neurol 35(3):201–208 26060899 10.1055/s-0035-1552617 PMC6413691

[4] Dekeyzer S, De Kock I, Nikoubashman O, Vanden Bossche S, Van Eetvelde R, De Groote J, Acou M, Wiesmann M, Deblaere K, Achten E (2017) Unforgettable – a pictorial essay on anatomy and pathology of the hippocampus. Insights Imaging 8(2):199–212 28108955 10.1007/s13244-016-0541-2 PMC5359145

[5] Eckstein K, Dymerska B, Bachrata B, Bogner W, Poljanc K, Trattnig S, Robinson SD (2018) Computationally efficient combination of multi-channel Phase Data from Multi‐echo acquisitions (ASPIRE). Magn Reson Med 79(6):2996–3006 29034511 10.1002/mrm.26963

[6] Fagan AJ, Bitz AK, Björkman-Burtscher IM, Collins CM, Kimbrell V, Raaijmakers AJE (2021) 7T MR Safety. J Magn Reson Imaging 53(2):333–346 32830900 10.1002/jmri.27319 PMC8170917

[7] Fervers P, Zaeske C, Rauen P, Iuga A-I, Kottlors J, Persigehl T, Sonnabend K, Weiss K, Bratke G (2023) Conventional and deep-learning-based Image reconstructions of Undersampled K-Space Data of the lumbar spine using compressed sensing in MRI: a comparative study on 20 subjects. Diagnostics 13(3):418 36766523 10.3390/diagnostics13030418 PMC9914543

[8] Gras V, Vignaud A, Amadon A, Le Bihan D, Boulant N (2017) Universal pulses: a new concept for calibration-free parallel transmission. Magn Reson Med 77(2):635–643 26888654 10.1002/mrm.26148

[9] Hagberg G, Bause J, Ethofer T et al (2017) Whole brain MP2RAGE-based mapping of the longitudinal relaxation time at 9.4T. NeuroImage 144:203–216 27663989 10.1016/j.neuroimage.2016.09.047

[10] Hangel G, Kasprian G, Chambers S et al (2024) Implementation of a 7T Epilepsy Task Force consensus imaging protocol for routine presurgical epilepsy work-up: effect on diagnostic yield and lesion delineation. J Neurol 271(2):804–818 37805665 10.1007/s00415-023-11988-5 PMC10827812

[11] Ivanov D, De Martino F, Formisano E et al (2023) Magnetic resonance imaging at 9.4T: the Maastricht journey. Magn Reson Mater Phys Biol Med 36(2):159–173 10.1007/s10334-023-01080-4 PMC10140139 37081247

[12] Kraff O, Orzada S (2023) Practical considerations on ultra-high field safety., pp 43–57

[13] Kraff O, Quick HH (2017) 7T: physics, safety, and potential clinical applications. J Magn Reson Imaging 46(6):1573–1589 28370675 10.1002/jmri.25723

[14] Martin P, Hagberg G, Loureiro J, Bause J, Lerche H, Focke N (2016) Can 9.4 T MRI improve lesion visualization in epilepsy patients? Magn Reson Mater Phys Biol Med 29(Supplement 1):S347–S348

[15] Nehrke K, Versluis MJ, Webb A, Börnert P (2014) Volumetric mapping of the brain at 7T using DREAM. Magn Reson Med 71(1):246–256 23413095 10.1002/mrm.24667

[16] Nguyen D, Sander J, Nguyen D, Lassonde M (2013) Prevalence of nonlesional focal epilepsy in an adult epilepsy clinic. Can J Neurol Sci 40(2):198–202 23419568 10.1017/s0317167100013731

[17] Opheim G, van der Kolk A, Bloch KM et al (2021) 7T Epilepsy Task Force Consensus recommendations on the Use of 7T MRI in Clinical Practice. Neurology 96(7):327–341 33361257 10.1212/WNL.0000000000011413 PMC8055334

[18] Pohmann R, Speck O, Scheffler K (2016) Signal-to-noise ratio and MR tissue parameters in human brain imaging at 3, 7, and 9.4 tesla using current receive coil arrays. Magn Reson Med 75(2):801–809 25820458 10.1002/mrm.25677

[19] Reeves C, Tachrount M, Thomas D et al (2016) Combined Ex vivo 9.4T MRI and quantitative histopathological study in normal and pathological neocortical resections in Focal Epilepsy. Brain Pathol 26(3):319–333 26268959 10.1111/bpa.12298 PMC4950048

[20] Shajan G, Kozlov M, Hoffmann J, Turner R, Scheffler K, Pohmann R (2014) A 16-channel dual‐row transmit array in combination with a 31‐element receive array for human brain imaging at 9.4 T. Magn Reson Med 71(2):870–879 23483645 10.1002/mrm.24726

[21] Sharma HK, Feldman R, Delman B, Rutland J, Marcuse LV, Fields MC, Ghatan S, Panov F, Singh A, Balchandani P (2021) Utility of 7 tesla MRI brain in 16 MRI negative epilepsy patients and their surgical outcomes. Epilepsy Behav Rep 15:100424 33521618 10.1016/j.ebr.2020.100424 PMC7820379

[22] Spencer D (2014) MRI (minimum recommended imaging) in Epilepsy. Epilepsy Curr 14(5):261–263 25346633 10.5698/1535-7597-14.5.261 PMC4189636

[23] Téllez-Zenteno J, Hernandez Ronquillo L, Moien-Afshari F, Wiebe S (2010) Surgical outcomes in lesional and non-lesional epilepsy: a systematic review and meta‐analysis. Epilepsy Res 89(2–3):310–318 20227852 10.1016/j.eplepsyres.2010.02.007

[24] Truong T-K, Clymer BD, Chakeres DW, Schmalbrock P (2002) Three-dimensional numerical simulations of susceptibility-induced magnetic field inhomogeneities in the human head. Magn Reson Imaging 20(10):759–770 12591571 10.1016/s0730-725x(02)00601-x

[25] Tse DHY, Poole MS, Magill AW, Felder J, Brenner D, Jon Shah N (2014) Encoding methods for B1 + mapping in parallel transmit systems at Ultra high field. J Magn Reson 245:125–132 25036294 10.1016/j.jmr.2014.06.006

[26] Tse DHY, Wiggins CJ, Ivanov D, Brenner D, Hoffmann J, Mirkes C, Shajan G, Scheffler K, Uludağ K, Poser BA (2016) Volumetric imaging with homogenised excitation and static field at 9.4 T. Magn Reson Mater Phys Biol Med 29(3):333–345 10.1007/s10334-016-0543-6 PMC4891373 26995492

[27] van Lanen RHGJ, Colon AJ, Wiggins CJ et al (2021) Ultra-high field magnetic resonance imaging in human epilepsy: a systematic review. Neuroimage Clin 30:102602 33652376 10.1016/j.nicl.2021.102602 PMC7921009

[28] van Lanen RHGJ, Wiggins CJ, Colon AJ et al (2022) Value of Ultra-high Field MRI in patients with suspected focal epilepsy and negative 3T MRI (EpiUltraStudy): protocol for a prospective, longitudinal, therapeutic study. Neuroradiology 64(4):753–764 34984522 10.1007/s00234-021-02884-8 PMC8907090

[29] van Osch MJP, Webb AG (2014) Safety of Ultra-high Field MRI: what are the specific risks? Curr Radiol Rep 2(8):61

[30] Wang Z, Alexopoulos A, Jones S, Jaisani Z, Najm I, Prayson R (2013) The pathology of magnetic-resonance‐imaging‐negative epilepsy. Mod Pathol 26(8):1051–1058 23558575 10.1038/modpathol.2013.52

[31] West S, Nevitt SJ, Cotton J, Gandhi S, Weston J, Sudan A, Ramirez R, Newton R (2019) Surgery for epilepsy. Cochrane Database Syst Rev 6(6):CD010541 31237346 10.1002/14651858.CD010541.pub3 PMC6591702

[32] Xu K, Xie P, Deng J et al (2023) Long-term ANT‐DBS effects in pilocarpine‐induced epileptic rats: a combined 9.4T MRI and histological study. J Neurosci Res 101(6):916–929 36696411 10.1002/jnr.25169

