# Supplementary material for: In vivo 9.4 Tesla MRI of a patient with drug-resistant epilepsy: Technical report
Source: Acta Neurochir (Wien). 2025 Jan 16;167(1):18. doi: 10.1007/s00701-024-06385-4 (PMC11739318; doi:10.1007/s00701-024-06385-4)
Supplement: Supplementary file 1 — (DOCX 1.43 MB) [file 701_2024_6385_MOESM1_ESM.docx]

**Supplementary Material**

**Supplementary material 1**

Scan parameters 3T MPRAGE (magnetization-prepared rapid gradient-echo) sagittal acquisition, TR (repetition time)/TE (echo time) 6.66/3ms, TI (inversion time) 917 ms, flip angle 8º, 0.9375 x 0.9375 x 0.9 mm voxel resolution, 210 slices, total acquisition time (TA) = 6:55 minutes.

Scan parameters 7T MP2RAGE sagittal acquisition TR/TE 5030/2.47 ms, TI 1/TI 2 900/2750 ms, flip angle 1/flip angle 2 5/3º, 0.7 mm isotropic voxel resolution, 240 slices, GRAPPA (GeneRalized Autocalibrating Partial Parallel Acquisition) factor 3, TA = 8.07 minutes. Dielectric pads were used.

Scan parameters 9.4T T1w MPRAGE sagittal acquisition, TR/TE 3750/3.64 ms, TI 1200 ms, flip angle 5º, 0.6 mm isotropic voxel resolution, 256 slices, GRAPPA factor 3, TA = 8:58 minutes

Scan parameters 9.4T 3D GRE (spoiled-gradient echo) ASPIRE[16], axial acquisition, TR = 33 ms, TE1/TE2/TE3/TE4 = 3.75/7.50/11.87/19.65 ms, flip angle 15º, 0.6 mm isotropic voxel resolution, 224 slices, GRAPPA factor 2, TA = 10:35 minutes; second 3D GRE ASPIRE, axial acquisition, TR = 27 ms, TE1/TE2/TE3/TE4 = 3.90/7.80/15.60/19.50 ms, flip angle 14º, 0.6 mm isotropic voxel resolution, 224 slices, TA = 10:35 minutes.

**Supplementary table 1.** Scan parameters of 3T, 7T and 9.4T T1 weighted imaging.

|  | **3T** | **7T** | **9.4T** |
| --- | --- | --- | --- |
| **Sequence** | MPRAGE | MP2RAGE | MPRAGE |
| **Head coil** | 32-channel | 32-channel | 31-channel |
| **Voxel size** | 0.9375 x 0.9375 x 0.9 | 0.7 x 0.7 x 0.7 | 0.6 x 0.6 x 0.6 |
| **Duration [m:s]** | 06:55 | 08:10 | 08:58 |
| **TR [ms]** | 2300 | 5030 | 3750 |
| **TE [ms]** | 2,32 | 2,47 | 3,64 |
| **TI [ms]** | 917 | 900 \| 2750 | 1200 |
| **Flip angle [°]** | 8 | 5 \| 3 | 5 |
| **No. of slices** | 210 | 240 | 256 |

**Supplementary material 2**

Apparent SNR and CNR was calculated for regions of interest, which were identified as: frontal (precentral gyrus/frontal lobe grey-white matter junction), insula, lateral temporal, and hippocampus, and compared between 3T, 7T and 9.4T. SNR was calculated for white and grey matter separately, except for the hippocampus which was calculated as a whole.

This analysis showed highest SNR values for the insula, temporal and hippocampal area at 3T, compared to 7T and 9.4T, while SNR was highest at 9.4T in the frontal area. A higher SNR was found at 9.4T compared to 7T, except for the temporal area which only slightly lower at 9.4T. CNR was quite similar across the different field strengths, as shown in Supplementary table 2.

**Supplementary table 2.** Signal-to-noise and contrast-to-noise ratios at 3, 7 and 9.4 Tesla for different brain areas.

|  | 3T MPRAGE | 7T MP2RAGE | 9.4T MPRAGE |
| --- | --- | --- | --- |
|  | Signal-to-noise ratio | | |
| Frontal white matter | 12.8 | 11.2 | 13.6 |
| Insula grey matter | 9.7 | 3.4 | 8.1 |
| Temporal white matter | 24.5 | 14.3 | 13.4 |
| Hippocampus | 10.8 | 3.2 | 5.6 |
|  | Contrast-to-noise ratio | | |
| Frontal | 3.2 | 5.0 | 3.6 |
| Insula | 2.7 | 3.2 | 3.2 |
| Temporal | 3.3 | 2.5 | 2.7 |

Notably, SNR in the temporal and hippocampal regions was higher at 3T in contrast to 7T and 9.4T, possibly due to signal attenuation in the inferior temporal lobes with increasing field strength. The shown apparent SNR and CNR values were not corrected for resolution. Therefore, since SNR and CNR generally decrease with smaller voxel size although partial volume effects may come in, the smaller voxel size at 7T and 9.4T show underappreciation of SNR and CNR values compared to 3T.[29, 30] Our analysis focused on SNR and CNR calculations within regions of interest relevant to epilepsy imaging, with SNR primarily emphasizing white matter regions because grey matter is thin and close to the coils, except for the more medial insular area.

**Supplementary material 3**


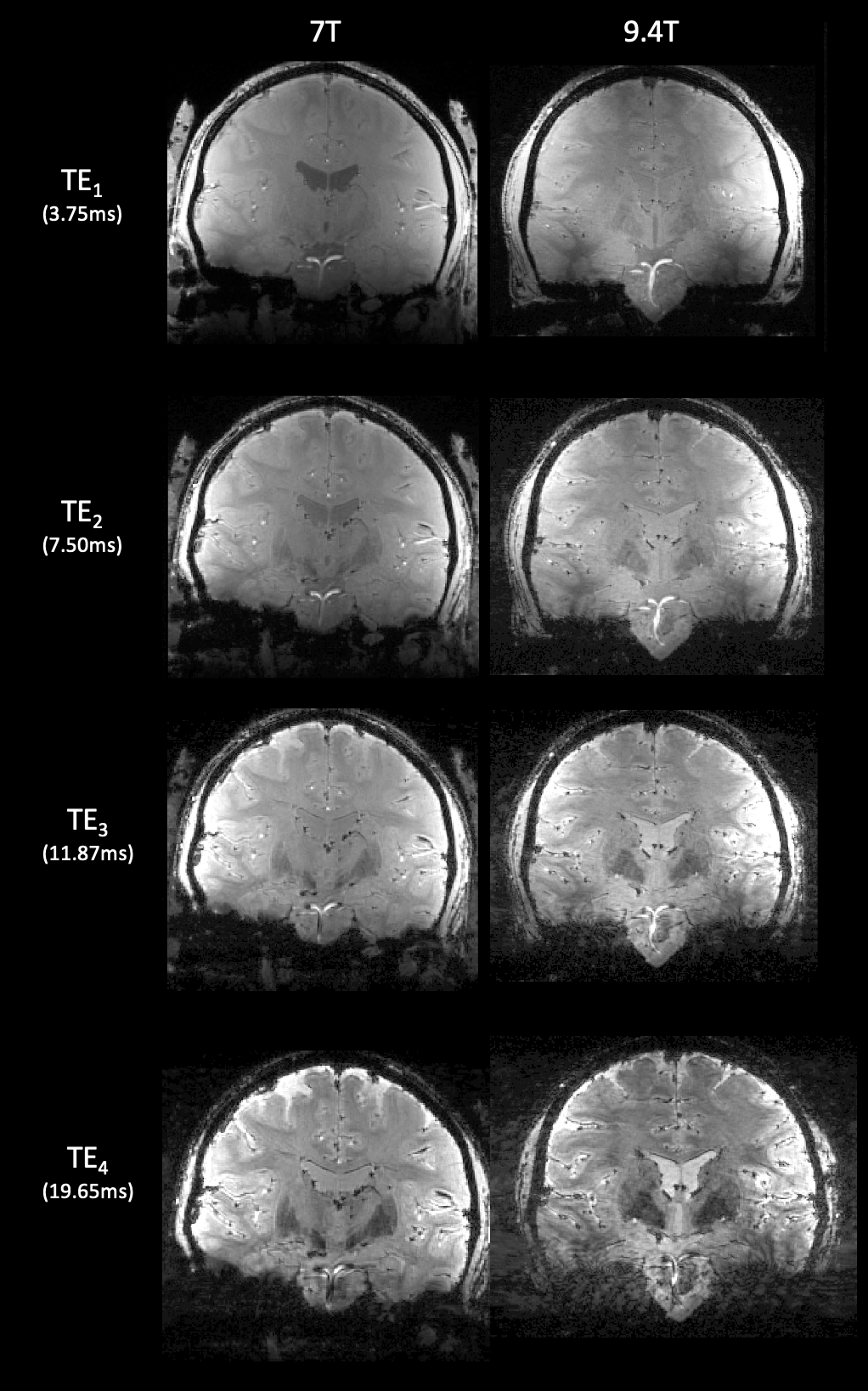


**Supplementary fig. 1** Comparison of the multi-echo gradient echo ASPIRE sequence between 7T and 9.4T at different echo times, showing differences in local contrast due to B1 field inhomogeneities, with stronger contrast in the basal ganglia due to shorter T2* at 9.4T compared to 7T. TE: echo time.

**
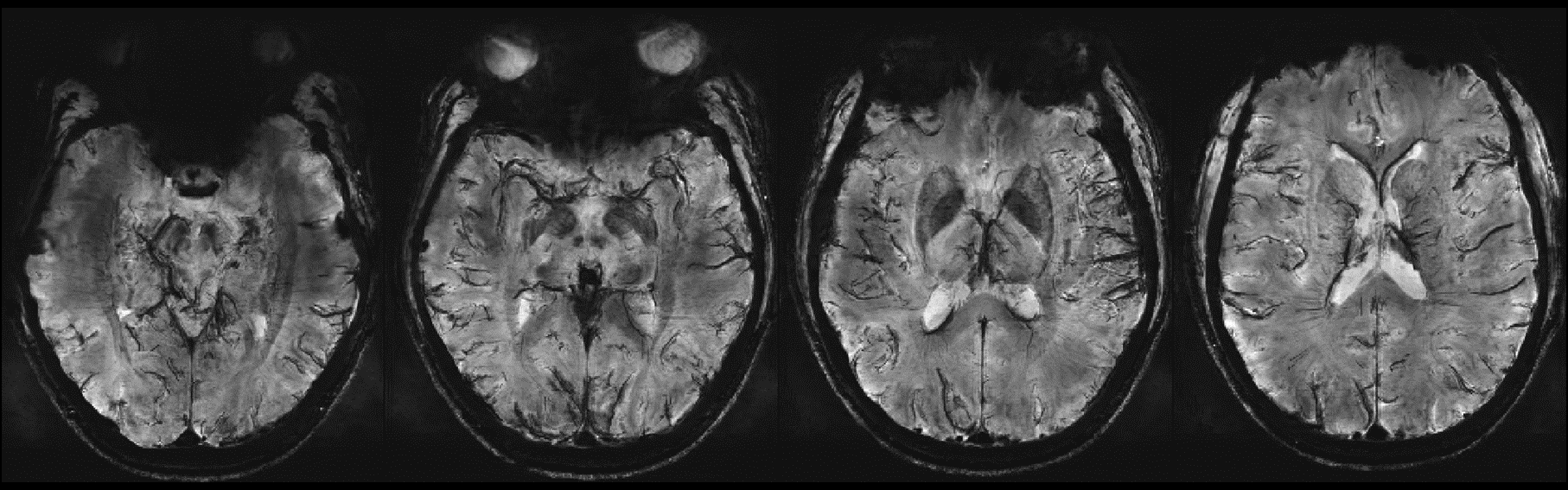
**

**Supplementary fig. 2** Axial susceptibility weighted imaging at 9.4T at different levels of the brain.
